# Ultraviolet Measurements and Photoclimatotherapy for Psoriasis at the Dead Sea: 25 Years of Experience

**DOI:** 10.3390/ijerph191912364

**Published:** 2022-09-28

**Authors:** Avraham I. Kudish, Efim G. Evseev, Guy Cohen, Marco Harari

**Affiliations:** 1 Blaustein Institutes for Desert Research, ED Bergmann Campus, Ben-Gurion University of the Negev, Beer Sheva 8410506, Israel; 2 The Dead Sea and Arava Science Center, Masada 86910, Israel; 3 Eilat Campus, Ben Gurion University of the Negev, Eilat 8855630, Israel; 4 DMZ Medical Center, Ein Bokek 86930, Israel

**Keywords:** ultraviolet irradiation, Dead Sea, psoriasis, photoclimatotherapy

## Abstract

Background: The Dead Sea basin is the lowest terrestrial site on the globe and is internationally recognized as a photoclimatotherapy center. Since the last century, questions were raised regarding a possible presence of unique incident ultraviolet irradiation, allowing the successful treatment of psoriasis, atopic dermatitis and other dermatological diseases. Aim: This research study aims to determine the characteristics of solar ultraviolet irradiation and to understand the mechanism of action of photoclimatotherapy while applying results to clinical protocols of treatments. Methods: A meteorological station was established at the Dead Sea basin to continuously measure global, UVB and UVA irradiation. The same irradiation parameters are also monitored continuously by a set of identical ultraviolet irradiation instruments installed on the campus of the Ben-Gurion University of the Negev in Beer Sheva. Results: This study details the results of these long-term measurements, as well as their correlation with the success obtained by clinicians treating psoriasis patients. Conclusions: A database of more than 25 years has enabled medical staff to establish tailor-made protocols for sun-exposure time intervals as a function of particular month and hour of day. The availability of such information significantly improved the results of photoclimatotherapy for psoriasis and simultaneously increased the safety of sun exposure at the Dead Sea.

## 1. Introduction

The Dead Sea is a terminal hypersaline lake located between the Judean mountains in Israel and the Moab mountains in Jordan. It is one of the saltiest bodies of water known, with approximately 345 g of mineral salts per liter [1,2]. It is situated at the lowest terrestrial site on earth, approximately 400 m below mean sea level. The Dead Sea region is recognized as a natural treatment site for patients with various skin disorders, such as psoriasis, atopic dermatitis, vitiligo and rheumatic and pulmonary diseases [3,4,5,6,7]. Although psoriatic patients have mainly been treated at the Deutsches Medizinisches Zentrum (DMZ medical center) [8] during the last 40 years, a substantial and accurate database on the Dead Sea therapeutic potential had been established. The success rate of the treatment protocol, measured on the basis of excellent to complete clearance after four weeks at the Dead Sea, exceeds 85% [5,9]. These clinical findings were presumed to be associated with a unique ultraviolet irradiation spectrum at the Dead Sea [10,11,12].

In the latter part of 1994, Professor Avraham Kushelevsky z”l and one of the present authors (A.I.K) were invited by the Regional R&D for the Dead Sea (now the “Dead Sea and Arava Science Center) to establish a meteorological station at the Dead Sea basin. The purpose of this study was to determine if the ultraviolet irradiation incident at the Dead Sea did indeed possess any unique properties that may contribute to the success of photoclimatotherapy in the treatment of psoriasis and/or other skin diseases at the Dead Sea. The results of this long-term research program and its correlation with the successful clinical outcome are now reported.

## 2. Materials and Methods

### 2.1. Site Parameters

The first step in initiating this research study was to establish a meteorological station at the Dead Sea basin to continuously measure global, UVB and UVA irradiation and other relevant bio-climatological parameters, viz., dry bulb temperature, barometric pressure and relative humidity. The Dead Sea meteorological station is located on the western shore of the Dead Sea at a site called Neve Zohar. The Neve Zohar site’s parameters are as follows: altitude of 375 m below the mean sea level, latitude 31°12′ N and longitude 35°22′ E. The same irradiation parameters were also monitored continuously by a set of identical ultraviolet irradiation instruments that were installed at a previous existing meteorological station located in Beer Sheva, on the campus of the Ben-Gurion University of the Negev, to provide a basis for inter-comparison. The site parameters for the Beer Sheva station are an altitude of 315 m above mean sea level, latitude 31°15′ N and longitude 34°45′ E. Beer Sheva is located in the southern Negev region of Israel, a semi-arid zone, at a distance of ca. 65 km to the west of the Dead Sea.

### 2.2. Broad-Band Measurements

The first phase of this study was to monitor global, UVB and UVA irradiation at both sites to determine if the ca., 700 m difference in altitude, viz., (Beer Sheva + 315 m) − (Neve Zohar − 375 m) = 690 m, results in significant irradiation intensity attenuation between the two sites.

The global irradiation at the Dead Sea station is measured by a Kipp & Zonen, CM 11 Pyranometer (Aix-en-Provence, France), whereas an Eppley Precision Spectral Pyranometer, Model PSP measures the global irradiation at Beer Sheva. The UV irradiation at both sites is monitored by the same instruments, viz., a Solar Light Co., Inc. (Glenside, PA, USA), Model 501A UV-Biometer to measure UVB and a Solar Light Co., Inc., analog UVA version of Model 501A UV-Biometer to measure UVA. Global, UVB and UVA irradiation meters are classified as broad-band instruments; viz., they measure the cumulative irradiation intensity on a horizontal surface within their respective spectral range. Campbell Scientific Instruments dataloggers are installed at each site (Model CR21 is installed at Neve Zohar and Model CR10 is installed at Beer Sheva) to monitor and store data at 10 min intervals (i.e., the instruments are scanned at 10 s intervals, and average values at 10 min intervals are calculated and stored).

The selection of this particular type of UV meter (biometer) was dictated in part by one of the aims of this study, i.e., to develop a database for monthly average daily and hourly UVB and UVA irradiation intensities to be utilized in the development of a photoclimatotherapy treatment protocol for psoriasis at the Dead Sea. UVB irradiation is reported in units of Minimum Erythema Dose per Hour (MED/h) for skin type 2, viz., the dose that causes minimal redness of the average skin type 2 after 1 h of exposure to the irradiation. The UVB meter measures the irradiation intensity and converts it to MED/h by cross-multiplying (programmed into the meter) the irradiating flux in the UVB spectral range and the Erythema Action spectra [13] (i.e., 1MED/h = 0.0583 W/m^2^ for a MED = 210 J/m^2^). The UVA meter measures the irradiating flux in the UVA spectral range in units of W/m^2^.

### 2.3. Narrow-Band Measurements

The second phase of this study was to determine if there was any significant selective attenuation within the UVB and/or UVA spectrum range. This was accomplished with the aid of a narrow-band spectrometer. Such measurements could not be carried out concurrently at the Dead Sea and Beer Sheva, since there was only a single spectroradiometer at our disposal. In order to overcome this problem, broad-band UVB and UVA meter measurements at both sites, Neve Zohar and Beer Sheva, were used to determine that the overall irradiation flux densities were of the same order of magnitude prior to carrying out an inter-comparison between the spectroradiometer measurements performed on two different but consecutive days. A narrow-band spectroradiometer, UV-Optronics 742, performed sporadic measurements, approximately once an hour, at both sites. They consisted of a scan from 295 to 380 nm at 1 nm intervals (the instrument’s band pass is 1.5 nm, as per the manufacturers’ specifications).

In addition, horizontal global irradiation intensities were compared at both sites. This parameter provides a more suitable criterion for the validation of the inter-comparison of the narrow-band spectra since they are minimally affected by the difference in optical path lengths as a result of the difference in altitudes between the sites. In other words, the degree of attenuation is inversely proportional to the wavelength, and the global irradiation is in the higher wavelength spectral range.

The Optronics 742 and all its peripheral equipment were transferred to the Dead Sea site (Neve Zohar) approximately once every two to three weeks for a day of measurements. The measurements consisted of a single scan per hour through the ultraviolet range (i.e., 295 to 380 nm) between 9:30 and 15:30 (Israel Standard Time). Identical sets of analyses were performed on several days, before and after the measurement day at Neve Zohar, at the Beer Sheva site in order to enhance the probability of obtaining two very similar days in order to perform an inter-comparison.

The theory of light scattering explains that the degree of attenuation of a solar ray is inversely proportional to its wavelength raised to some power, i.e., scattering caused by the following:

Air molecules: Rayleigh scattering is proportional to λ^−4^;

Water molecules: The empirical scattering coefficient is proportional to ~λ^−2^;

Aerosols: The empirical scattering coefficient [14] is proportional to ~λ^−0.75^.

The extent of attenuation by any type of scattering is, a priori, a function of optical path length. The object of these measurements was to determine if the ca. 700 m difference in altitude between the two sites is sufficient for causing a significant difference between the UVB and UVA spectra at the two sites.

### 2.4. Additional Measurements

Several additional measurements, which provided more information regarding the quality of the ultraviolet irradiation environment at the Dead Sea basin, were performed using a Solar Light Co., Inc. Microtops II, Ozone Monitor-Sunphotometer. This portable device contains three narrow-band light filters that measure UVB irradiation intensities at three different wavelengths within the UVB spectrum, viz., 305.5 ± 0.3, 312.5 ± 0.3 and 320.0 ± 0.3 nm. Microtops II was designed to be used to measure the stratospheric ozone layer thickness, which is determined as a function of the relative intensities of the three narrow-band UVB readings. This is accomplished by utilizing a unique built-in programmed algorithm. It was adapted to the current study because the 305 nm irradiation is within the peak erythemal spectral range and the 312 nm irradiation is within the psoriasis therapeutic wavelengths. Consequently, Microtops II provides information regarding the relative intensity of the erythemal to therapeutic irradiation as a function of time of day.

## 3. Results

The ultraviolet B irradiation database consists of measurements initiated in February 1995, whereas that of UVA comprised measurements from June that year. UVB and UVA irradiation intensities are continuously monitored, with the exception of periodic scheduled annual factory calibration by the manufacturer (Solar Light Co., Inc.) and interruptions resulting from power failures. The two meteorological stations are part of the Israel Meteorological Service’s national network.

### 3.1. Broad-Band Measurements

**Figure 1:**
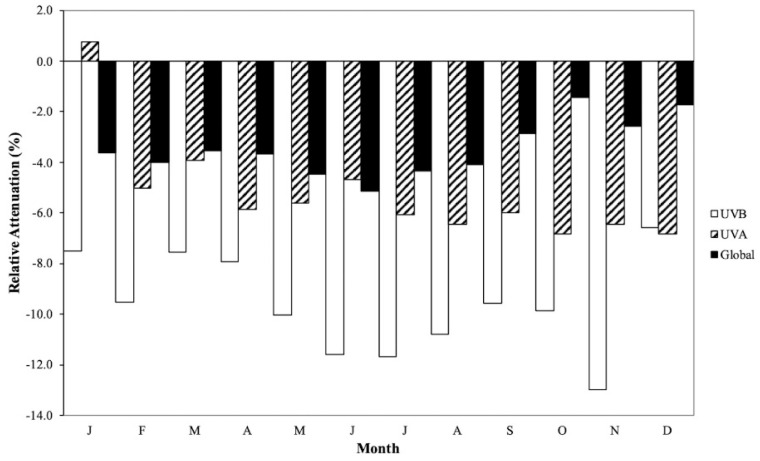
Monthly average daily % relative attenuation of irradiation intensities of global, UVB and UVA irradiation: [%(Neve Zohar − Beer Sheva)/Beer Sheva].

The results of the broad-band irradiation measurements during the time period from January 1996 to June 2019 are reported as monthly average daily values in Table 1, Table 2 and Table 3 for global, UVB and UVA. The % relative attenuation is defined as follows:

(X_Neve Zohar_ − X_Beer Sheva_) × 100/X_Beer Sheva_ (1)

where X is denotes broad-band irradiation, and it is is shown in Figure 1.

A priori, the broad-band irradiation is expected to be lower at the Dead Sea compared to Beer Sheva due to their ca. 700 m difference in altitude, viz., its longer optical path length and, consequently, greater probability of undergoing scattering phenomena. The actual additional optical path length, ∆(OPL), due to the lowest altitude, which a solar ray has to traverse, is given by the following:

∆(OPL) = change in altitude/cos θ_z_, (2)

where θ_z_ is the solar zenith angle at which the solar ray subtends with the normal to the surface. Consequently, θ_z_ decreases and its cosine increases, approaching unity with increasing solar altitudes, i.e., the height of the sun in the sky. This is why the magnitude of the solar irradiation intensity peaks during the summer months: The solar rays have to traverse a shorter optical path length through the atmosphere. The increased attenuation during irradiation intensities as the time from solar noon increases towards sunrise or sunset also a function of the optical path’s length. In addition, it also is the reason for why the UVB index peaks at midday on clear days, viz., the shortest daily optical path length.

It should be emphasized, once again, that such scattering is inversely proportional to the wavelength; it is the greatest for UVB and lowest for global irradiation. In addition, the monthly relative attenuation for a particular type of irradiation at two different sites is influenced by particular climatic conditions, i.e., different microclimates at each site. The Judean mountain range located west of the Dead Sea basin prevents winter rain clouds emanating from the Mediterranean Sea from depositing rain in the Dead Sea basin. Consequently, the Dead Sea basin is characterized by a much clearer sky relative to Beer Sheva during the rainy season (December to March).

The above is corroborated by the relative magnitudes of the monthly average daily global radiation measured at both sites: With the exception of the month of June, the relative attenuation values are less than 5%, cf. Table 1. They vary between a maximum of -5.13% during June and a minimum of −1.71% during December.

The % relative attenuation of the monthly daily UVB irradiation values between the Dead Sea and Beer Sheva is significantly greater than the corresponding values for the UVA irradiation, cf. Table 2 and Table 3. This is attributed to the fact the attenuation by scattering phenomena is inversely proportional to the wavelength, and the UVB spectrum is the lowest wavelength range incident on Earth. Once again, the seasonal variation in the magnitude of the % relative attenuation is a function of the microclimates at the two sites.

The % relative attenuation in the case of UVB irradiation varies between a maximum in August of −11.68% and a minimum in December of −6.56%, whereas the corresponding values for UVA irradiation are −6.84% in December and +0.77% in January. It should be noted that with the exception of January, all other values for the relative attenuation of UVA irradiation are negative: in the range between −6.84% in December and −3.94% in March.

The results from broad-band ultraviolet irradiation measurements, as discussed above and reported in Table 2 and Table 3, exhibit the attenuation of both UVB and UVA irradiation incidents at the Dead Sea basin relative to Beer Sheva. It is observed from an inter-comparison of the two tables that UVB irradiation is attenuated to a significantly greater extent than UVA irradiation. This in itself is to be expected since, as mentioned previously, the degree of attenuation caused by scattering phenomena is inversely proportional to the wavelength. Consequently, there would be no explicit advantage to photoclimatotherapy at the Dead Sea relative to other sites; the sun exposure time required for the treatment of psoriasis would have to be longer in order to achieve the required dose of UVB irradiation. On the other hand, there would be a distinct advantage to psoriatic patients undergoing photoclimatotherapy at the Dead Sea if the spectral rays within the erythema range were reduced to a greater extent than those within the therapeutic beneficial spectrum [15].

### 3.2. Narrow-Band Measurements

**Figure 2:**
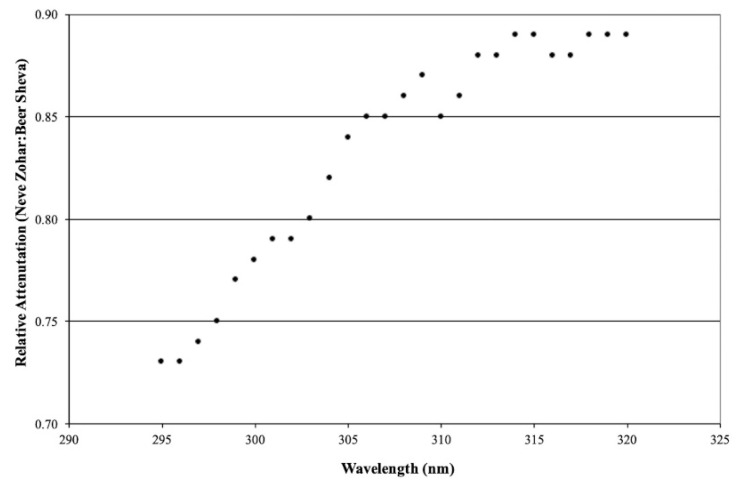
Relative UVB irradiation reduction within its spectral range: (Neve Zohar:Beer Sheva).

**Figure 3:**
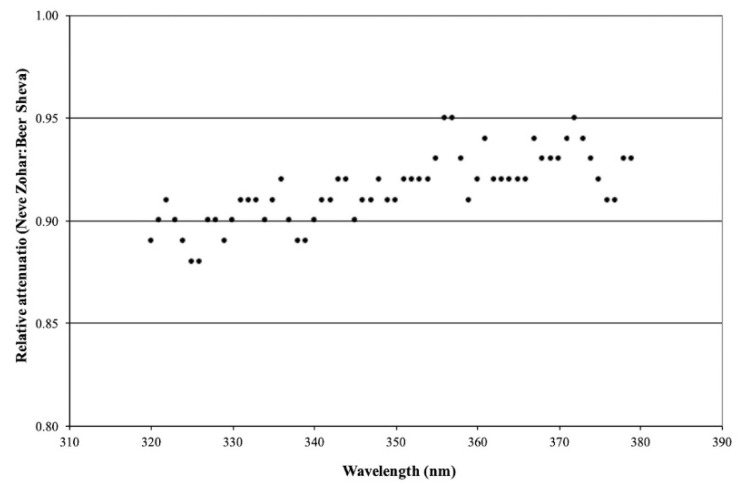
Relative UVA irradiation attenuation within its spectral range: (Neve Zohar:Beer Sheva).

Sporadic measurements with UV-Optronics 742 (a narrow-band spectroradiometer; scans from 295 to 380 nm, 1 nm intervals) were performed at both sites. Results from this phase of the study for the UVB (295–320 nm) and UVA (320–380 nm) spectral ranges are shown in Figure 2 and Figure 3, respectively. The data presented in these figures are from measurements performed during August on two consecutive days, i.e., a single day of measurements at each site. They are representative of the measurements performed during this study.

Once again, the criteria to justify the inter-comparison of the measurements at the two sites on consecutive days are based mainly on the relative magnitude of the horizontal global irradiation intensities measured at the two sites, viz., their approach to unity. The graphs illustrate the ratio of the irradiation intensities at the two sites as a function of wavelength throughout UVB and UVA spectral ranges. A significant spectral selectivity was observed for UVB irradiation, with the degree of attenuation decreasing as the wave-length increased. In the range of the peak erythema action spectra, ca. 300 nm, the degree of attenuation is between 0.73 and 0.79 (27% and 21%), whereas in the spectral range of the UVB beneficial to psoriasis, ca. 312 nm, the degree of attenuation is between 0.85 and 0.89 (15% and 11%).

In the case of UVA, the degree of irradiation mitigation is significantly less than that for the UVB irradiation and its spectral selectivity was much less obvious, varying between 0.89 and 0.95 (11% and 5%). The scatter observed in the measured data is not unexpected, considering the very low irradiation intensities measured by the spectroradiometer. It is important to emphasize that these observations are in complete agreement with the theory of light scattering; viz., the degree of attenuation by light scattering phenomena is inversely proportional to its wavelength.

It is this spectral selectivity within the UVB spectrum, i.e., the greater degree of attenuation within the erythema spectral range relative to that in the psoriasis therapeutic spectral range that makes photoclimatotherapy at the Dead Sea so attractive. Consequently, patients undergoing this treatment are exposed to a lower cumulative dose of erythema irradiation during their stay at the Dead Sea.

### 3.3. Additional Measurements

The Microtops II, Ozone Monitor-Sunphotometer was utilized to measure relative irradiation intensities as a function of time at wavelengths of 305.5 nm and 312.5 nm, i.e., the relative irradiation intensities within the erythema to the psoriasis therapeutic spectra.

**Figure 4:**
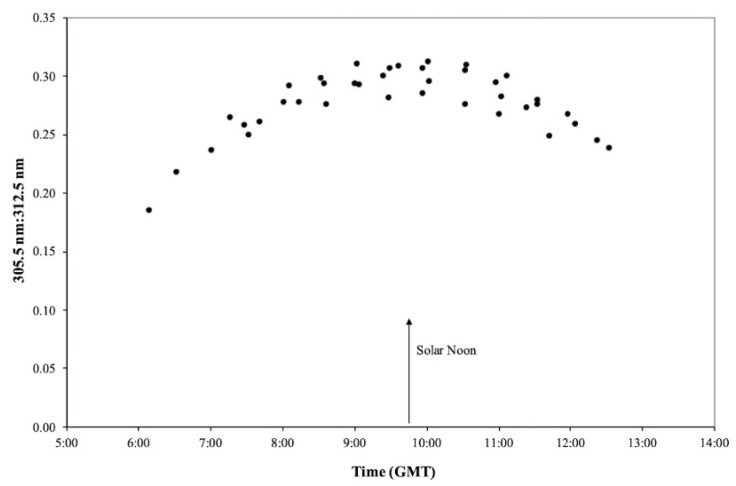
Ratio of 305.5:312.5 nm irradiation intensities at Neve Zohar during the July as a function of the time of day (Israel Standard Time = GMT +2).

The results of such measurements performed at the Dead Sea during July, as a function of time, are shown in Figure 4. The Israel Standard Time is equivalent to GMT +2 h and during July solar time precedes local time by about 15 min. Consequently, the shortest optical path length during the month of July occurs at ~09:45 GMT, as evidenced by the minimum attenuation at 305.5 nm relative to 312.5 nm irradiation intensities, viz., the relative maximum ratio of 305 nm to 312 nm.

The optical path length for a solar ray varies diurnally and attains its daily minimum at solar noon, and it increases towards sunrise and sunset. The extent of attenuation of the ultraviolet irradiation and resultant spectral selectivity varies similarly, i.e., a daily minimum at solar noon. Consequently, in order to minimize exposure to erythema irradiation, patient sun exposure should be avoided around midday hours.

### 3.4. Application to Dead Sea Psoriasis Treatment Protocol

The results of broad-band measurements are utilized to develop sun-exposure timetables as a function of patient skin type and the month of treatment in order to optimize the psoriasis treatment protocol. The goal is to make sure that the patient receives the proper cumulative dose of therapeutic UVB irradiation during his treatment while minimizing his exposure to the erythema irradiation.

The sun-exposure timetables are based upon the following:

The UVB irradiation intensity equivalent of an MED as a function of skin type as defined by COST-713 [16], cf. Table 4;
The recommended UVB irradiation dose required during a patient’s stay at the Dead Sea, e.g., Table 5 for a 2-week stay based upon clinical experience at the Dead Sea Clinic, Ein Bokek (Dead Sea basin);
Combining the data from Table 4 and Table 5 together with UVB broad-band measurements generates monthly average hourly UVB irradiation doses available (MED) according to skin type at the Dead Sea, e.g., Table 6.

Tables such as Table 6 are developed for each skin type. The hours on a table are divided into three groups based upon the monthly average hourly UVB irradiation intensity:

Hours suitable for sun exposure (less then 2 MED/h)—italics;
Hours not suitable for sun exposure due to insufficient irradiation intensity—underlined;
Hours not suitable for sun exposure because of the relatively high irradiation intensity—bold.

The next phase is to generate a patient sun-exposure timetable in accordance with skin types and the month and duration of stay at the Dead Sea. An example of such a sun-exposure timetable is shown in Table 7 for a 2-week stay during the month of March at the Dead Sea for a patient with a skin type 2.

The prescribed cumulative UVB irradiation dose is defined in accordance with Table 5, which in the case of a skin type 2 is 25.5 MED or 637.5 mJ/cm^2^. This dosage is achieved by gradually increasing the daily dosage of UVB irradiation from an initial 0.4 MED (10 mJ/cm^2^) on day 1 to 3.4 MED (85.0 mJ/cm^2^) on day 14. The sun-exposure time intervals for the month of March are listed in columns four and five and are based upon monthly hourly data reported in Table 6. From day 1 to day 6, the patient can choose between either morning or afternoon sun-exposure time intervals, whereas from day 7 to day 14, both morning and afternoon sun-exposure time intervals are required to achieve the prescribed daily dosage.

The patient’s total sun-exposure time during a 2-week stay at the Dead Sea will be between 27.0 and 28.5 h depending upon the preference for either morning or afternoon sun exposure between day 1 and day 6.

### 3.5. Photoclimatotherapy Results

Psoriasis is a chronic inflammatory skin disease for which its manifestations are sharply demarcated, erythematous, pruritic plaques covered in silvery scales. The disease has strong genetic predisposition intertwined with environmental triggers. In a recently published review on psoriasis therapies, the authors highlighted the Dead Sea as the most common destination for climatotherapy [16]. Out of a total of 34 papers cited in the section on “*Balneotherapy and Climatotherapy*”, 18 studies were related to the Dead Sea treatment. Excellent outcomes were reported in almost all of these publications.

**Figure 5:**
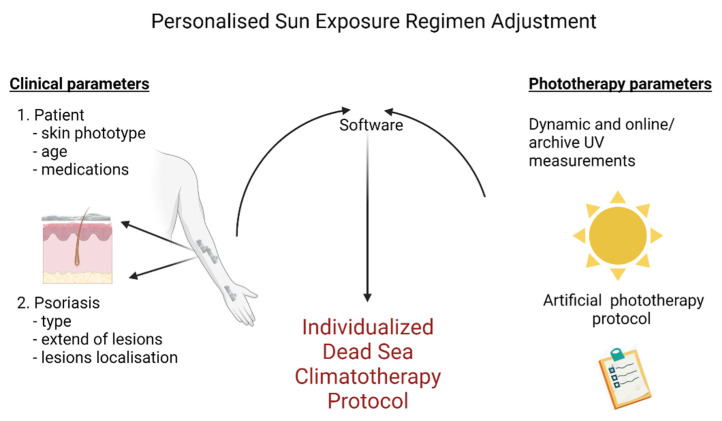
Dead Sea personalized climatotherapy protocol. Created with BioRender.com.

The authors, however, reported an ambiguity concerning the recommendations for sun exposure time intervals. Indeed, a 1996 publication recommended only 3 h per day, while another and recommended up to 7 h per day the following year. As we have shown in previous paragraphs, the use of on-site UVB measurements defines the daily exposure time interval required (summarized in Figure 5). Consequently, there is no longer need for approximation; viz., the doses of solar photoclimatotherapy are calculated precisely and transformed into exposure time intervals for each patient.

It should be noted that two important topics were not included in the abovementioned review. First, the efficacy and safety of Dead Sea photoclimatotherapy has been verified many times but its influence on the quality of life of patients has often been neglected. A clinical study has demonstrated the positive influence on the quality of life of patients who underwent photoclimatotherapy at the Dead Sea sustained for several months [9]. Second, studies on the cost-effectiveness of psoriasis treatments only rarely mention the superiority of phototherapy and, in particular, that of photoclimatotherapy. In chronic and “relapsing” diseases, economic evaluation necessitates the determination of remission time in order to draw firm conclusions regarding the relative superiority of a particular therapy.

Conclusion regarding the long-term results of Dead Sea Photoclimatotherapy have recently been published [17]. The authors reported data of 17 patients who underwent treatment at the Dead Sea and were followed up at home. In a commentary [18] on this article, one of the present authors (MH) emphasized the difficulty to arrive at any conclusions on the basis of such a small sample size. Based upon our years of experience treating psoriasis at the Dead Sea, we assess the remission phase to exceed 6 months. Nevertheless, we agree that further clinical studies on the long-term effectiveness of photoclimatotherapy at the Dead Sea should be performed.

Some will say that Dead Sea Photoclimatotherapy is an example of personalized medicine. Indeed, sun exposure times can vary during the same day and for the same disease depending on the patient’s clinical parameters. However, even if individual parameters and measurements of irradiance at the Dead Sea are the basis of our recommendations (cf., Figure 5), we believe that we are still far from the current definition of personalized medicine. In fact, we are only giving an adequate dosage of ultraviolet irradiation as we would for antibiotic treatments. According to published definitions [19], precision medicine prescribes much more. Personalized medicine improves patient care by using biological and molecular information from diseases, genetic and proteomics as well as metabolomics. Actually, our research center pursues this goal mainly through the study of the skin microbiome [20] and an improved understanding of the disease’s symptoms [21].

In conclusion, clinicians and patients have greatly benefited from solar spectrum analyses conducted at the Dead Sea. Applying the UVB database has made it possible to transform an empirical natural treatment into a well-codified and scientifically based therapy.

### 3.6. Ongoing Research Program

A major inconvenience of photoclimatotherapy in general and at the Dead Sea in particular, is that it requires exposing the skin and, thus, the patient to the sun for extended periods. This can be quite uncomfortable for the patient undergoing the treatment. The hypothesis, on which the following research program is based, is that the wavelength range of UVB irradiation spectra is the shortest of all terrestrial solar irradiation and, as a result, will be most highly scattered; i.e., a high percentage of it will diffuse. Solar or global irradiation comprises two components:

Beam or direct irradiation—rays come directly from sun and are considered to have both a single point source and direction;

Diffuse irradiation—rays are scattered as they traverse the atmosphere and have neither a defined source nor direction.

Consequently, if the UVB irradiation is found to be highly scattered at the Dead Sea as a result of it being the lowest terrestrial site on earth, viz., longer optical path length during optimal sun-exposure time intervals, there is no need for the patient to be directly exposed to sun.

There are two options to determine the composition of incident solar irradiation: the beam and diffuse fractions:

Measure the global irradiation on a horizontal surface and the normal incident beam irradiation by means of a measuring instrument that tracks the sun on its daily east to west (sunrise to sunset) path and with intermittent adjustments for its changes in solar altitude (north to south seasonal track);

Measure the global irradiation on a horizontal surface using two identical measuring instruments: one to measure global irradiation and the second is outfitted with a shading device that blocks out the beam’s irradiation and thereby measures the diffuse fraction.

**Figure 6:**
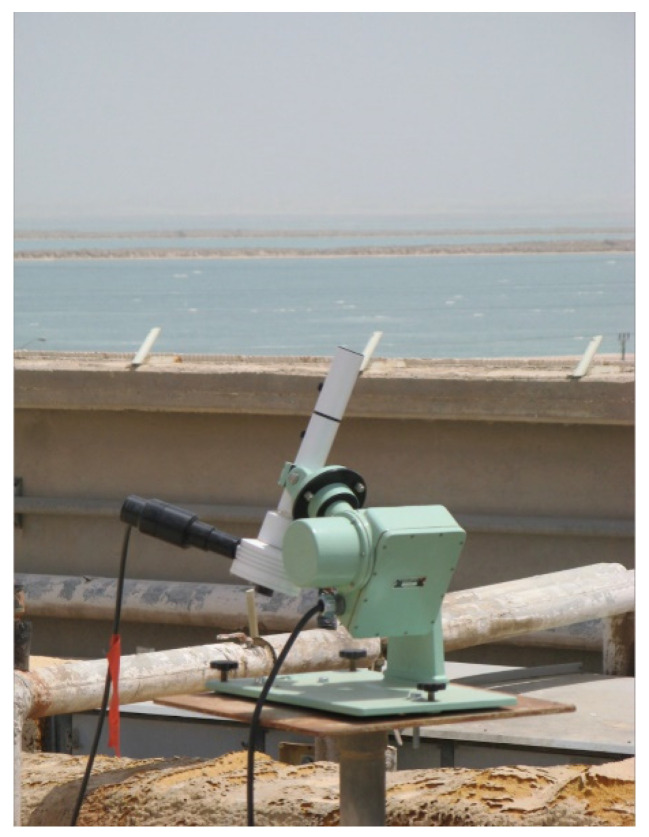
The normal incidence UVB measured by means of a Model 501A UV-Biometer mounted on an Eppley Solar Tracker Model St-1.

It was decided in this study to use the first option because it is a direct measurement of the normal incident beam irradiation, whereas the second option entails a number of correction factors in order to arrive at the value for the true diffuse irradiation. In order to accomplish this, it was necessary to acquire such an instrument. A prototype tracking instrument composed of a Model 501A UV-Biometer mounted on an Eppley Solar Tracker Model St-1 was specially designed for this project by Solar Light Company Inc. to measure the normal incident UVB beam irradiation incident at the Dead Sea basin, cf. Figure 6. The beam and diffuse fractions of the incident UVB irradiation on a horizontal surface are determined by concurrently measuring the UVB global irradiation and the normal incident UVB beam irradiation by means of the following equations:

I_UVB,d_ = I_UVB,g_ − I_UVB,b,h_, (3)

I_UVB,b,h_ = I_UVB,b,n_(cosθ_z_) (4)

where I_UVB,d_ is the UVB diffuse irradiation on a horizontal surface, I_UVB,g_ is the UVB global irradiation on a horizontal surface, I_UVB,b,h_ is the hourly UVB irradiation on a horizontal surface, I_UVB,b,n_ is the hourly normal incident UVB beam irradiation and θ_z_ is the solar zenith angle.

The results of that study have been published recently [22], and the diffuse fraction throughout the year at the Dead Sea was observed to exceed 80%. The application of the results of these measurements suggests a possible revision of the photoclimatotherapy treatment protocol for psoriasis patients at the Dead Sea’s medical spas.

It may be possible to take advantage of this relatively high diffuse fraction by allowing patients to receive their daily dose of UVB irradiation without direct exposure to the sun, i.e., to receive their daily dose of UVB irradiation while positioned under a sunshade. This would necessitate an increase in sun-exposure time intervals by approximately 20-25%, since UVB irradiation intensities beneath a sunshade are less than that on an exposed surface.

The preliminary clinical testing of this hypothesis with respect to the photoclimatotherapy of psoriasis patients, as well as in other skin disorders, at the Dead Sea is promising. This revised photoclimatotherapy protocol will allow the psoriasis patient to take advantage of the unique diffuse fraction of UVB irradiation and increase the patient’s compliance to the updated sun-exposure protocol, beneath sunshades, for somewhat longer time periods but for the same irradiation dose and concomitant therapeutic action.

The conclusions of the study regarding the high diffuse fraction of UVB irradiation are also relevant with regard to sun exposure in general [23]. Shading devices such as an umbrella or horizontal overhang/shade provide relief from the solar global radiation but provide limited protection from the diffuse fraction of UVB irradiation. This is especially true at locations such as beaches where the incident irradiation is highly reflected by the sand and a significant fraction of the solar global UVB irradiation does penetrate this supposedly ‘protective or comfort zone’ under the shade device. Consequently, it is imperative to minimize sun-exposed body surfaces by wearing adequate clothing or using sunscreen even when under such shade devices.

## 4. Conclusions

The Dead Sea basin’s unique climatic conditions are due to both its latitude and low altitude, which is approximately 400 m below sea level. They include high barometric pressure as a result of it being the lowest terrestrial point on the Earth and, consequently, increased oxygen partial pressure, high and stable temperatures, low humidity, a paucity of rainfall and a unique spectral composition of the incident ultraviolet solar irradiation.

The unique ultraviolet spectral composition is the result of the relatively longer optical path length that the solar rays must pass through the Earth’s atmosphere prior to being incident on the surface, i.e., the lowest terrestrial point on the Earth. As it traverses the atmosphere, solar irradiation undergoes attenuation by scattering due to air molecules, water vapor and aerosols, and the attenuation is inversely proportional to the wavelength raised to a power. Consequently, ultraviolet irradiation processes possessing the shortest wavelength are attenuated to the greatest extent, and UVB is attenuated more than UVA irradiation processes. In fact, erythema UV irradiation is attenuated to the greatest extent, since it is located at the lower end of the UVB spectrum. This combination of solar, climatic and balneotherapeutic factors present at the Dead Sea is unequaled elsewhere in the world.

Exposure to the sun and, in particular, the spectral composition of the solar ray’s incident on the ground at the Dead Sea area have been proven to be highly effective in temporarily suppressing the clinical features of psoriasis, viz., achieving remission. Broad-band measurements of the solar UVB and UVA irradiation at the Dead Sea relative to identical measurements at Beer Sheva (315 m above mean sea level) exhibit a general attenuation of both UVB and UVA irradiation intensities at the Dead Sea basin; UVB irradiation was attenuated to a much greater extent than UVA irradiation.

It was also observed that the relative irradiation intensities within the UVB spectrum between the erythema and therapeutic spectral ranges are also a function of the time of day. Therefore, patient sun-exposure regimens should be scheduled for early morning hours or late afternoon hours in order to limit their exposure to harmful erythema rays, which peak during midday hours, and this also avoids being outdoors during very hot midday hours [23,24].

Pertinent conclusions based upon the analysis of the concurrent monitoring of the solar UVB and UVA at the Dead Sea and Beer Sheva, initiated in 1995, can be summarized as follow [24]:

UVA and UVB solar irradiation intensities at the Dead Sea basin are both attenuated relative to Beer Sheva, with UVB at a greater extent than UVA;
The degree of attenuation is inversely proportional to the wavelength. As a result, the erythema wavelength range (ca. 300 nm) is attenuated to greatest extent than the therapeutic wavelength range for psoriasis (ca. 311 nm) at the Dead Sea;
The diurnal optical path length is at the minimum at solar noon. Consequently, solar exposure should be avoided because the therapeutic-to-erythema ratio is also a minimum;
The incident UVB solar irradiation at the Dead Sea has a higher ratio of therapeutic-to-erythema irradiation relative to other sites.

Based upon the medical literature and the clinical results obtained during our 25 years of experience applying Dead Sea photoclimatotherapy for psoriasis, we expect both excellent short-term and good long-term success with respect to such a treatment.

## Figures and Tables

**Table 1:** Monthly average daily % relative attenuation of global irradiation intensities (kW/m^2^).

Month	Neve Zohar (kW/m^2^)	Days	Beer Sheva (kW/m^2^)	Relative Attenuation (%) ^1^
Jan	2.98	692	3.10	−3.64
Feb	3.82	675	3.98	−4.02
Mar	5.10	755	5.29	−3.53
Apr	6.36	700	6.60	−3.67
May	7.32	739	7.66	−4.48
June	7.92	698	8.35	−5.13
July	7.70	733	8.06	−4.36
Aug	7.09	707	7.39	−4.08
Sept	6.11	710	6.28	−2.86
Oct	4.72	727	4.79	−1.43
Nov	3.57	631	3.67	−2.55
Dec	2.94	677	2.99	−1.71

^1^ % Relative attenuation = (Global_Neve Zohar_ − Global_Beer Sheva_) × 100/Global_Beer Sheva_.

**Table 2:** Monthly percent daily average relative reduction in UVB irradiation intensities (MED).

Month	Neve Zohar (MED)	Days	Beer Sheva (MED)	Relative Attenuation (%) ^1^
Jan	6.00	509	6.49	−7.51
Feb	9.01	463	9.96	−9.53
Mar	13.22	649	14.29	−7.53
Apr	17.31	700	18.80	−7.92
May	21.18	740	23.54	−10.03
June	24.57	701	27.79	−11.59
July	23.60	702	26.72	−11.68
Aug	21.28	678	23.86	−10.82
Sept	17.35	657	19.19	−9.56
Oct	11.75	683	13.04	−9.89
Nov	7.47	566	8.58	−13.00
Dec	5.46	537	5.84	−6.56

^1^ % Relative attenuation = (UVB_Neve Zohar_ − UVB_Beer Sheva_) × 100/UVB_Beer Sheva_.

**Table 3:** Monthly percent daily average relative reduction in UVA irradiation intensities (W/m^2^).

Month	Neve Zohar (W/m^2^)	Days	Beer Sheva (W/m^2^)	Relative Attenuation (% ) ^1^
Jan	140.28	494	139.21	0.77
Feb	175.35	468	184.58	−5.00
Mar	234.56	614	244.19	−3.94
Apr	288.56	641	306.50	−5.85
May	340.76	688	360.93	−5.59
June	381.38	681	400.17	−4.70
July	363.90	694	387.40	−6.07
Aug	330.90	659	353.80	−6.47
Sept	279.05	658	296.80	−5.98
Oct	210.13	694	225.57	−6.84
Nov	155.39	631	166.13	−6.46
Dec	124.89	588	134.06	−6.84

^1^ % Relative attenuation = (UVA_Neve Zohar_ − UVA_Beer Sheva_) × 100/UVA_Beer Sheva_.

**Table 4:** MED values per skin type as defined by COST-713 [16].

Skin Type	1 MED
I	20 mJ/cm^2^
II	25 mJ/cm^2^
III	35 mJ/cm^2^
IV	45 mJ/cm^2^

**Table 5:** Recommended cumulative UVB irradiation dose during a 2-week stay at the Dead Sea *.

Skin Type	MED’s	mJ/cm^2^
II	25.5	637.5
III	27.5	962.5
IV	33.5	1507.5

* Based upon clinical experience at the Dead Sea Clinic, Ein Bokek (Dead Sea basin).

**Table 6:** Monthly average hourly UVB irradiation dose available (MED) for Skin Type 2 at the Dead Sea.

Month/hour	7/8	8/9	9/10	10/11	11/12	12/13	13/14	14/15	15/16	16/17
**Mar.**	0.25	*0.67*	*1.22*	*1.70*	*1.94*	*1.86*	*1.48*	*0.95*	0.46	0.14
**Apr.**	0.48	*1.02*	*1.65*	**2.18**	**2.41**	**2.27**	*1.83*	*1.20*	0.60	0.19
**May**	*0.70*	*1.37*	**2.07**	**2.60**	**2.81**	**2.64**	**2.14**	*1.44*	*0.76*	0.29
**June**	*0.81*	*1.56*	**2.35**	**2.97**	**3.22**	**3.04**	**2.49**	*1.72*	*0.97*	0.41
**July**	*0.71*	*1.40*	**2.18**	**2.78**	**3.06**	**2.94**	**2.44**	*1.71*	*0.96*	0.40
**Aug.**	*0.61*	*1.27*	**2.02**	**2.63**	**2.89**	**2.76**	**2.23**	*1.49*	*0.79*	0.29
**Sept.**	0.48	*1.08*	*1.77*	**2.30**	**2.51**	**2.31**	*1.76*	*1.08*	0.50	0.14
**Oct.**	0.30	*0.37*	*1.26*	*1.66*	*1.78*	*1.58*	*1.13*	*0.62*	0.24	0.03

**Table 7:** Recommended sun-exposure protocol for skin type 2 for the month of March for a 2-week stay at the Dead Sea; Days 1–6 either morning or afternoon; Days 7–14 both morning and afternoon.

Day	MED	mJ/cm^2^	Morning (hours)	Afternoon (hours)
1	0.4	10.0	8:00–8:35	14:35–15:00
2	0.6	15.0	8:00–8:55	14:20–15:00
3	0.8	20.0	8:00–9:05	14:10–15:00
4	1.1	27.5	8:00–9:20	13:55–15:00
5	1.3	32.5	8:00–9:30	13:45–15:00
6	1.5	37.5	8:00–9:40	13:40–15:00
7	1.7	42.5	8:00–9:10	14:05–15:00
8	1.9	47.5	8:00–9:15	14:00–15:00
9	2.1	52.5	8:00–9:20	13:55–15:00
10	2.3	57.5	8:00–9:25	13:50–15:00
11	2.5	62.5	8:00–9:30	13:50–15:00
12	2.8	70.0	8:00–9:35	13:40–15:00
13	3.1	77.5	8:00–9:45	13:35–15:00
14	3.4	85.0	8:00–9:50	13:30–15:00
Total	25.5	637.5		
Subtotal exposure	Days 1–6 Days 7–14	Either/or Both	425 min (7.1 h) 710 min (11.8 h)	335 min (5.6 h) 575 min (9.6 h)
Total exposure	Maximum Minimum	1710 min (28.5 h) 1620 min (27.0 h)	(28.5 h) (27.0 h)	

## Data Availability

All data are available from authors upon request.
